# High Fat-to-Muscle Ratio Was Associated with Increased Clinical Severity in Patients with Abdominal Trauma

**DOI:** 10.3390/jcm12041503

**Published:** 2023-02-14

**Authors:** Jiang Li, Fengchan Xi, Yuanchen He, Chuanrui Sun, Wenkui Yu, Xiling Wang

**Affiliations:** 1 Key Laboratory of Public Health Safety, Ministry of Education, School of Public Health, Fudan University, Shanghai 200231, China; 2 Research Institute of General Surgery, Affiliated Jinling Hospital, Medical School of Nanjing University, Nanjing 210093, China; 3 Department of Intensive Care Unit, Affiliated Drum Tower Hospital, Medical School of Nanjing University, Nanjing 210008, China

**Keywords:** abdominal trauma, obesity, obesity paradox, body composition, fat-to-muscle ratio

## Abstract

Overweight and moderate obesity confer a survival benefit in chronic diseases such as coronary artery disease and chronic kidney disease, which has been termed the “obesity paradox”. However, whether this phenomenon exists in trauma patients remains controversial. We performed a retrospective cohort study in abdominal trauma patients admitted to a Level I trauma center in Nanjing, China between 2010 and 2020. In addition to the traditional body mass index (BMI) based measures, we further examined the association between body composition-based indices with clinical severity in trauma populations. Body composition indices including skeletal muscle index (SMI), fat tissue index (FTI), and total fat-to-muscle ratio (FTI/SMI) were measured using computed tomography. Our study found that overweight was associated with a four-fold risk of mortality (OR, 4.47 [95% CI, 1.40–14.97], *p* = 0.012) and obesity was associated with a seven-fold risk of mortality (OR, 6.56 [95% CI, 1.07–36.57], *p* = 0.032) compared to normal weight. Patients with high FTI/SMI had a three-fold risk of mortality (OR, 3.06 [95% CI, 1.08–10.16], *p* = 0.046) and double the risk of an intensive care unit length of stay ≥ 5 d (OR, 1.75 [95% CI, 1.06–2.91], *p* = 0.031) compared to patients with low FTI/SMI. The obesity paradox was not observed in abdominal trauma patients, and high FTI/SMI ratio was independently associated with increased clinical severity.

## 1. Introduction

Obesity has become a global epidemic, with 39% of adults being overweight and 13% being obese, respectively [1]. Obesity is a well-established risk factor for multiple chronic health conditions including diabetes, hypertension, dyslipidemia, arthritis, heart disease and certain malignancies [2,3]. Despite this, the obesity paradox, which refers to the phenomenon whereby a decreased mortality is present in overweight and moderate obesity compared to normal weight patients, has been observed in various acute and chronic diseases such as sepsis, acute respiratory distress syndrome, coronary artery disease and chronic kidney disease [4,5,6,7]. However, the impact of obesity on trauma patients remains controversial. Hakam et al. and Dvorak et al. have observed the obesity paradox defined by body mass index (BMI) in general trauma patients using the US National Trauma Data Bank (NTDB) [8,9]. However, Cone et al. failed to observe this phenomenon in patients with chest and head trauma using the US Trauma Quality Improvement Program (TQIP) database [10,11]. In the case of abdominal trauma, abdominal adipose, as a direct traumatized organ, might pose more severe metabolic disturbances compared with injuries in other body regions [12].

Nevertheless, BMI is an imprecise measure of adiposity, which fails to differentiate between fat and muscle mass or to quantify adiposity distribution, and patients with the same BMI may have different body composition phenotypes [13,14,15]. It is reported that both low skeletal muscle mass or sarcopenia and high fat mass increased the risk of poor prognosis in trauma patients [16,17]. However, few studies evaluated the combined effects of muscle and fat mass on the clinical outcomes of trauma patients. The fat-to-muscle ratio, a novel index for evaluating the combined effects of muscle and fat mass, has been demonstrated to be associated with a higher risk of metabolic syndrome in healthy adults and a higher risk of mortality in patients with chronic kidney disease [18,19]. Specifically, adults with the highest tertile of fat-to-muscle ratios had a four times higher risk of metabolic syndrome compared with those with the lowest tertile of fat-to-muscle ratios. Hemodialysis patients with higher fat-to-muscle ratios had a three times higher risk of all-cause death than those with lower fat-to-muscle ratios.

However, to our knowledge, the associations between fat-to-muscle ratio and clinical outcomes in trauma patients remain unclear. Studying the relationships between the fat-to-muscle ratio and clinical outcomes would help to take target interventions based on individual body composition at an early stage to improve prognosis. Hence, our study aimed to examine whether the obesity paradox exists in patients with abdominal trauma, and if the fat-to-muscle ratio is independently associated with clinical severity in abdominal trauma patients.

## 2. Materials and Methods

This study followed the Strengthening the Reporting of Observational Studies in Epidemiology (STROBE) reporting guideline checklist [20].

### 2.1. Study Design and Patients

**Figure 1:**
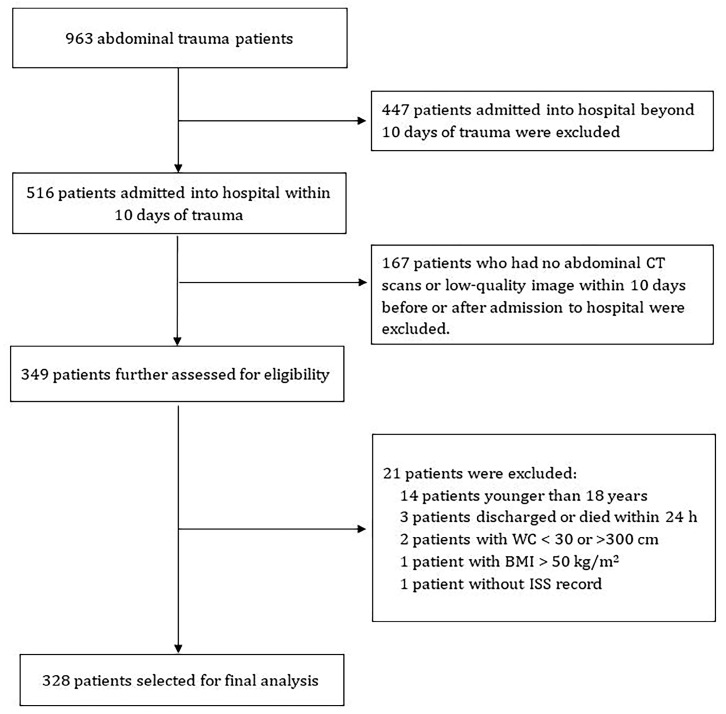
Flow chart of the included participants. CT, computed tomography; WC, waist circumference; BMI, body mass index; ISS, injury severity score.

We conducted a retrospective cohort study in abdominal trauma patients admitted to the General Surgical Department of the Jinling Hospital Affiliated with Nanjing University Medical School between January 2010 and March 2020. Our hospital has a national Level I abdominal trauma center and is one of the initiating units of the Jiangsu Trauma Treatment Alliance, which could provide high quality data for our research. Abdominal trauma was defined as a blunt or penetrating injury to the abdominal cavity, which is located between the thorax cephalad and the pelvis caudad. Specifically, the upper limit is represented by a horizontal plane passing through the base of the xiphoid appendix and the spinous process of the 12th dorsal vertebra, and the lower limit starts from the pubic symphysis, passes through the entire inguinal arc and iliac crest, and terminates at the spinous process of the 5th lumbar vertebra [21]. During the study period, 963 consecutive patients were admitted to our hospital after abdominal trauma. Among these patients, 516 patients who were admitted to the hospital within 10 days of trauma met the inclusion criteria (Figure 1). After excluding 167 patients who had no abdominal computed tomography (CT) scans or low-quality images within 10 days before or after admission, 349 patients were further assessed for eligibility. Finally, a total of 328 patients were included in the study after excluding 14 patients younger than 18 years, 3 patients who were discharged or died within 24 h, 1 patient without an injury severity score (ISS) record, 1 patient with BMI above 50 kg/m^2^ and 2 patients with waist circumference (WC) below 30 or above 300 cm.

### 2.2. Data Collection

The following variables were extracted from the electronic medical record: demographic characteristics (age, sex, smoking history, alcohol consumption, hypertension, diabetes, height, weight, WC), clinical parameters (Glasgow Coma Scale (GCS), systolic blood pressure (SBP), heart rate (HR), respiratory rate (RR), trauma mechanism, body-region specific abbreviated injury scale (AIS), ISS, laparotomy), and outcomes of mechanical ventilation, death within 28 days of admission, length of stay in hospital and intensive care unit (ICU). Weight, height, and WC were measured on admission for each patient. BMI (defined as the weight in kilograms divided by the square of the height in meters) of less than 18.5 kg/m^2^, BMI of 18.5–23.9 kg/m^2^, BMI of 24.0–27.9 kg/m^2^ and BMI of 28 kg/m^2^ or higher were defined as underweight, normal weight, overweight and obese, respectively, according to the criteria of the Working Group on Obesity in China (WGOC) for adults [22].

### 2.3. CT-Based Body Composition Assessment

**Figure 2:**
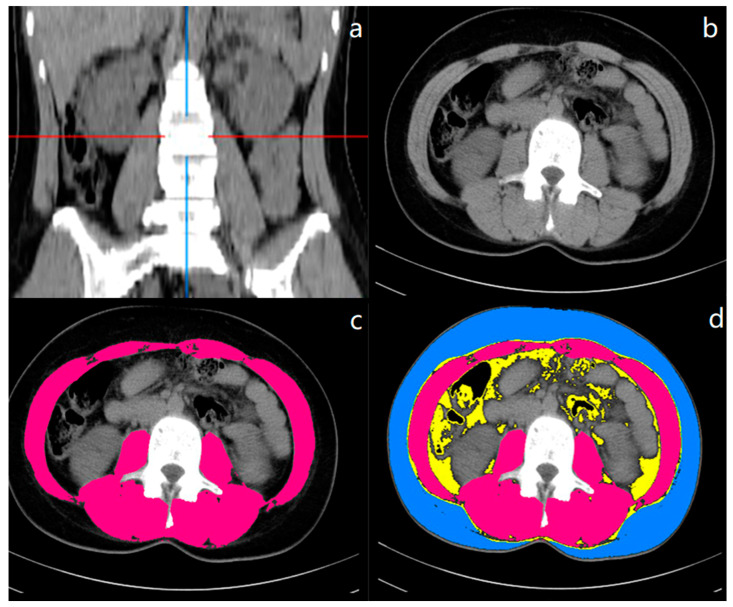
CT measurement of body composition at the level of the third lumbar vertebra (L3). (**a**) represented the coronal image and the red and blue lines identified the level of L3 and median coronal dividers, respectively. (**b**–**d**) represented the cross-sectional images. In the (**c**,**d**) images, different body composition areas were distinguished by different colors (blue: subcutaneous adipose tissue area; red: skeletal muscle area; the yellow area within the inner wall of the muscle: visceral adipose tissue area).

Body composition indices were measured using contrast-enhanced or unenhanced CT scans at the level of the third lumbar vertebra (L3). The image quality was evaluated by three independent radiologists, who were blinded to all patient information using a 5-point Likert scale with 5 being excellent [23]. All CT scans of acceptable quality were then transferred to the Image J2 software (The National Institutes of Health, Washington, MD, USA) to measure skeletal muscle and adipose tissue areas. The tissue cross-sectional areas were analyzed using tissue-specific Hounsfield Units (HU) attenuation ranges: (1) −29 and +150 HU for skeletal muscle, (2) −150 to −50 HU for viscera adipose tissue, and (3) −190 to −30 HU for subcutaneous and intramuscular adipose tissue [24] (Figure 2). The outer contour of the abdominal wall was automatically delineated and the abdominal muscular wall was manually delineated. Total muscle area (TMA), subcutaneous fat area (SFA) and visceral fat area (VFA) were measured. Total fat area (TFA) was the sum of SFA and VFA.

Body composition indices were then normalized by the square of height (cm^2^/m^2^) [25,26]. We used TMA, TFA, SFA, and VFA to calculate the skeletal muscle index (SMI, TMA [cm^2^]/height^2^ [m^2^]), fat tissue index (FTI, TFA [cm^2^]/height^2^ [m^2^]), subcutaneous adipose tissue index (SATI, SFA [cm^2^]/height^2^ [m^2^]), visceral adipose tissue index (VATI, VFA [cm^2^]/height^2^ [m^2^]). We further calculated fat-to-muscle area ratios including the total fat-to-muscle area ratio (FTI/SMI), subcutaneous fat-to-muscle area ratio (SATI/SMI), and visceral fat-to-muscle area ratio (VATI/SMI) to assess the combined effects of fat and skeletal muscle on abdominal trauma patients [27]. In addition, the visceral-to-subcutaneous fat area ratio (VATI/SATI) was computed to evaluate the impact of fat distribution on trauma patients [28]. All these indices were divided into low and high groups according to sex-specific median cutoff values [29].

### 2.4. Outcomes

The primary clinical outcome was 28-day mortality. Secondary clinical outcomes included the use of mechanical ventilation during hospitalization and an ICU length of stay ≥ 5 d.

### 2.5. Statistical Analysis

Kendall’s coefficient of concordance (Kendall’s W) and intraclass correlation coefficient (ICC) (0 indicates no agreement between raters; 1 indicates perfect agreement between raters) were calculated to assess the consistency of CT image quality assessment among the three radiologists. The normality of distribution was assessed using the Shapiro–Wilk test. Continuous data with a normal distribution were expressed as the means (standard deviations, SDs) and compared by *t*-test or one-way analysis of variance, as appropriate. Continuous data without a normal distribution were expressed as the medians (interquartile ranges, IQRs) and compared by Mann–Whitney U test or Kruskal–Wallis test as appropriate. Categorical data were expressed as frequencies and percentages and compared by χ^2^ test or Fisher exact test as appropriate. Correlations between body composition indices were assessed by Pearson correlation analyses.

The associations of the BMI category and fat-to-muscle area ratios with 28-day mortality, mechanical ventilation, and ICU length of stay ≥ 5 d were assessed with univariable and multivariable logistic regression analyses, respectively. We adjusted for potential confounders including age, sex, ISS ≥ 16, hypertension, diabetes, smoking history, alcohol consumption, HR > 120 beats/min, RR > 20 beats/min, SBP < 90 mmHg, GCS < 9 and laparotomy. These variables were chosen based on their clinical relevance to outcomes, and the previous literature [10,11].

In our dataset, there were 7 (2.1%) patients with unknown history of smoking and alcohol consumption and 1 (0.3%) patient without SBP measurement at admission. Missing values were replaced by the mean value for the continuous variable and by the mode (i.e., negative) for the categorical variables, including smoking and alcohol consumption history. All statistical tests were two-sided, and *p* < 0.05 was considered statistically significant. All analyses were conducted using R version 4.1.1. (R Foundation for Statistical Computing, Vienna, Austria).

## 3. Results

There was a strong consistency in the evaluation of CT image quality between three radiologists, indicated by 0.836 of Kendall’s W (*p* < 0.001) and 0.806 of ICC (*p* < 0.001). The median age was 43 (31–53) years and 266 (81.1%) were males (Table 1). A total of 301 (91.8%) patients suffered from blunt trauma and most patients were severely injured, with 241 (73.5%) of patients having an ISS ≥ 16. 13 (4.0%), 233 (71.0%), 60 (18.3%) and 22 (6.7%) patients were classified as underweight, normal weight, overweight and obese, respectively. The median length of ICU stay was 5 (2–10) days. The need for mechanical ventilation occurred in 101 (30.8%) patients, and 21 (6.4%) patients died within 28 days of admission.

**Figure 3:**
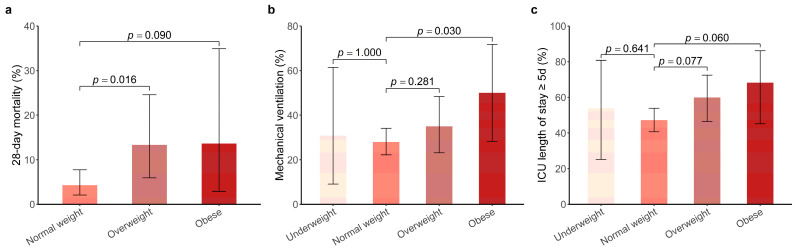
Comparison of clinical severity including 28-day mortality (**a**), mechanical ventilation (**b**) and ICU length of stay ≥ 5 d (**c**) in abdominal trauma patients with different BMI classifications. *p* values above each bar plot indicate significance between the respective BMI group of abdominal trauma patients (underweight, overweight, and obese) and the normal weight. The error bars denote the confidence interval, which was calculated based on exact binomial distribution. ICU, intensive care unit.

FTI/SMI, VATI/SMI, and SATI/SMI ratios increased significantly with higher BMI classes (Table 1). Patients with higher classes of BMI had more severe injuries, particularly in the abdomen, indicated by a higher proportion of ISS ≥ 16 and AIS score abdomen ≥ 3 in patients with overweight (88.3% and 81.7%, respectively) and obesity (86.4% and 95.5%, respectively). Patients with overweight (13.3% vs. 4.3%, *p* = 0.016) and obesity (13.6% vs. 4.3%, *p* = 0.090) had higher 28-day mortality compared with normal weight patients (Table 1, Figure 3). Patients with overweight and obesity were more likely to require mechanical ventilation (35.0% vs. 27.9%, *p* = 0.281 and 50.0% vs. 27.9%, *p* = 0.030, respectively) and to stay in the ICU longer than 5 days (60.0% vs. 47.2%, *p* = 0.077 and 68.2% vs. 47.2%, *p* = 0.060, respectively). In the multivariable logistic model, after adjusting for all potential confounders we found that the risk of death within 28 days was around 4.5 times (OR, 4.47 [95% CI, 1.40–14.97]; *p* = 0.012) and 6.6 times (OR, 6.56 [95% CI, 1.07–36.57]; *p* = 0.032) higher in patients with overweight and obesity than normal weight patients (Table 2). However, such an association was not observed between overweight or obesity and mechanical ventilation, and ICU length of stay ≥ 5 d.

**Figure 4:**
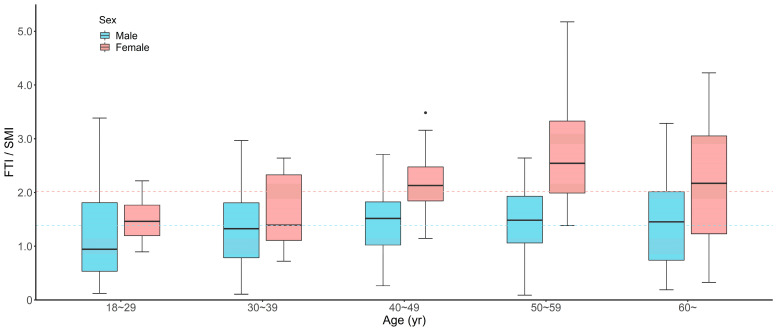
Box plots showing differences in FTI/SMI according to sex and age. The blue and red dashed lines represent the median FTI/SMI for male (1.39) and female (2.02) patients, respectively. FTI, fat tissue index; SMI, skeletal muscle index.

**Figure 5:**
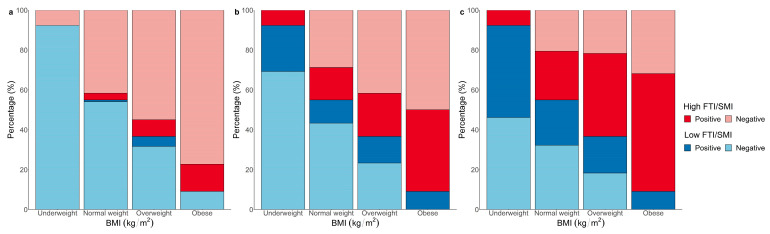
Bar plots showing the percentage of patients with combinations of low or high FTI/SMI ratios and corresponding positive or negative clinical outcomes in each BMI group. (**a**): Mortality within 28 days; (**b**): Mechanical ventilation; (**c**): ICU length of stay ≥ 5 d. BMI, body mass index; FTI, fat tissue index; SMI, skeletal muscle index.

Patients with high FTI/SMI ratios were more likely to be older (46 vs. 40 years, *p* = 0.001) compared with low FTI/SMI ratios patients (Table 1). In patients of all ages, females have significantly higher FTI/SMI ratios compared with males (2.02 vs. 1.39, *p* < 0.001) (Figure 4). After further stratifying patients by age groups, in general, FTI/SMI increased with age and females tended to have higher FTI/SMI across all age groups. Patients with high FTI/SMI ratios had a higher proportion of alcohol consumption (33.5% vs. 22.0%, *p* = 0.019), with higher prevalence of diabetes (10.4% vs. 3.7%, *p* = 0.017) and hypertension (23.2% vs. 14.0%, *p* = 0.033) (Table 1). Moreover, these patients also had higher BMI (22.9 vs. 21.7 kg/m^2^, *p* < 0.001) and a higher proportion of ISS ≥ 16 (79.3% vs. 67.7%, *p* = 0.017). For clinical outcomes, the use of mechanical ventilation (37.2% vs. 24.4%, *p* = 0.012) and death within 28 days (9.8% vs. 3.0%, *p* = 0.013) occurred in a significantly higher percentage in patients with high FTI/SMI ratios than in those with low FTI/SMI ratios. Patients with high FTI/SMI ratios also tended to have longer ICU lengths of stay (6 vs. 4 days, *p* = 0.009). When we stratified patients according to BMI, we found that one (7.7%) patient with underweight, 105 (45.1%) patients with normal weight, 38 (63.3%) patients with overweight and 20 (90.9%) patients with obesity had high FTI/SMI ratios (Figure 5). Furthermore, we found that, among those normal weight patients, eight (79.1%) of the deaths, 38 (58.4%) of the mechanical ventilation users and 57 (51.8%) of ICU stays > 5 d occurred in the high FTI/SMI subgroup.

In multivariable analyses for evaluating the associations between fat-to-muscle area ratios and clinical outcomes, we found that the risk of death within 28 days and ICU length of stay ≥ 5 d was about 3.1 (OR, 3.06 [95% CI, 1.08–10.16]; *p* = 0.046) and 1.8 (OR, 1.75 [95% CI, 1.06–2.91]; *p* = 0.031) times higher in high FTI/SMI ratios patients than low FTI/SMI ratios patients (Table 2). After distinguishing adipose tissues in different depots, we found that a high SATI/SMI ratio significantly increased the risk of death within 28 days (OR, 3.33 [95% CI, 1.16–11.13]; *p* = 0.034), while a high VATI/SMI ratio (OR, 1.99 [95% CI, 1.05–3.82]; *p* = 0.037) significantly increased the risk of mechanical ventilation. However, there was no significant association between ICU length of stay and the VATI/SMI (OR, 1.39 [95% CI, 0.83–2.33]; *p* = 0.215) or SATI/SMI ratio (OR, 1.28 [95% CI, 0.79–2.10]; *p* = 0.321).

## 4. Discussion

Our study examined whether overweight and obesity confer a survival benefit in abdominal trauma patients and comprehensively assessed the association between skeletal muscle and fat mass and clinical severity by using the fat-to-muscle ratio. We found that patients with overweight and obesity have higher mortality rates than normal weight patients, which indicated that the obesity paradox defined by BMI might not be applied to patients with abdominal trauma. A higher FTI/SMI ratio was independently associated with an increased risk of 28-day mortality and an ICU length of stay ≥ 5 d.

The obesity paradox has been demonstrated in multiple acute and chronic diseases, probably because overweight or moderate obesity was associated with higher energy reserves, anti-inflammatory immune profile, and inflammatory preconditioning, which conferred a survival benefit [4]. Inflammatory preconditioning refers to the fact that increased baseline inflammation caused by obesity triggers multiple anti-inflammatory and antioxidant endogenous pathways to counteract new onset acute inflammatory reactions in critical illness [30,31]. The anti-inflammatory immune profile refers to the anti-inflammatory adipokine profile and M2-type macrophage accumulation in the chronic phase of critical illness [32]. However, the obesity paradox remains conflicting in trauma patients. Dvorak et al. and Farhat et al. have observed this phenomenon in general trauma patients [8,9], while Cone et al. failed to observe it in patients with severe blunt chest trauma and head trauma [10,11]. In the present study, the obesity paradox was not observed in abdominal trauma patients, either. These conflicting results could be attributed to the high heterogeneity of the trauma population and the methodological limitations of using BMI as a proxy for obesity. Abdominal trauma patients have different trauma mechanisms and injury locations, and most of them present with multiple injuries. Delayed diagnosis and treatment were common in this specific trauma population due to their complexity and concealment [33]. Furthermore, several studies have found that the obesity paradox existed in patients with cancer or heart failure when using BMI but it disappeared when using body composition or WC [34,35]. Therefore, more accurate measures to better characterize body composition and fat distribution were warranted instead of BMI alone [4].

CT imaging is easily available in clinical practice and has been evaluated as an accurate method to evaluate skeletal muscle and fat mass. Thus, we used L3 level CT to provide precise estimates of muscle and adipose tissue that are highly correlated with the whole-body volumes of muscle and adipose [36]. Prior studies have examined the associations of skeletal muscle mass and fat mass individually with clinical outcomes in trauma patients, however, their findings were controversial. For skeletal muscle mass, several studies have demonstrated that low skeletal muscle mass was an independent predictor of poor prognosis in patients with trauma [16,37], but this failed to be validated in other studies [38,39]. For fat mass, Shashaty et al. have reported that abdominal adiposity was independently associated with acute kidney injury in critically ill trauma patients [17]. However, no significant associations were observed between fat mass and other clinical outcomes including mortality and hospital or ICU length of stay in other studies [37,38].

Skeletal muscle mass and fat mass should be considered together to avoid the above contradictory results, due to the close correlation between skeletal muscle mass and body fat mass. In this study, there was a positive relationship between FTI and SMI (*r* = 0.319, *p* < 0.001) (Supporting information Figure S1), so the assessment of the effect of either SMI or FTI required the simultaneous consideration of both [19].

Therefore, we used the fat-to-muscle ratio to evaluate the effect of the combined muscle and fat mass on abdominal trauma patients. Given gender differences in the FTI/SMI ratio, we grouped patients as high FTI/SMI and low FTI/SMI ratios according to sex-specific medians. The average FTI/SMI were represented as medians in view of non-normality, while as means in a study of trauma patients in Germany [38]. By comparison, we found that males in our study have lower FTI/SMI (1.39 vs. 1.47) but females have higher FTI/SMI (2.02 vs. 1.83), which demonstrated differences in body architecture and body composition among different populations. The fat-to-muscle ratio could be an indicator of sarcopenic obesity, which referred to high fat mass accompanied by low muscle mass. Sarcopenic obesity has been shown as a predictor of metabolic syndrome and cardiovascular diseases, and poor prognosis in patients undergoing hemodialysis [18,19,27]. In our study, we also found that patients with high FTI/SMI ratios had a higher prevalence of diabetes and hypertension, and a high FTI/SMI ratio was significantly associated with poor clinical outcomes after abdominal trauma. There are several biological mechanisms to explain the observed association. Skeletal muscle plays an important role in regulating immune function, glucose disposal and protein synthesis [37]. Therefore, sarcopenic patients have a worse recovery after trauma due to their decreased resilience and physiological reserve [16]. Moreover, obesity is a metabolic syndrome involving hypertension, hyperglycemia, dyslipidemia and a pro-inflammatory state, which has been reported to increase the risk of adverse outcomes after trauma. Specifically, adipose tissue participates in the systematic proinflammatory response to acute trauma, which produces numerous inflammatory mediators such as circulating interleukin-6 (IL-6), tumor necrosis factor-α (TNF-α), and leptin [40].

In addition, we explored the combined effect of skeletal muscle mass and fat mass in different depots; however, the results were not consistent for different outcome measures. The SATI/SMI ratio was associated with 28-day mortality, and the VATI/SMI ratio was associated with the use of mechanical ventilation, but neither was associated with the length of ICU stay. The reasons for the apparent differences in these associations were not clear, but may be related to the different effects of visceral and subcutaneous adipose tissue distribution. Two prior studies have shown that visceral to subcutaneous adipose tissue distribution was not associated with increased inflammatory profiles or clinical outcomes after trauma [40,41]. However, a recent study found that lower visceral to subcutaneous adipose tissue distribution was associated with increased inflammatory response and worse clinical outcomes in multiple trauma patients [28]. In this study, we have examined whether body fat distribution by calculating the VATI/SATI ratio has an impact on abdominal trauma patients as well, but no significant associations were found between the VATI/SATI ratio and clinical outcomes (Supporting information Table S1). Therefore, the effect of fat distribution on outcomes in trauma patients needs to be further explored.

Our findings have important clinical implications. Given that muscle and fat are intricately correlated, monitoring the configuration of individual body composition by using the FTI/SMI ratio is critical to improving the prognosis in patients with abdominal trauma. Those patients with high FTI/SMI ratios based on sex-specific medians (1.39 for males, 2.02 for females) may benefit from early appropriate nutritional, pharmacological and exercise interventions. In addition, it is worth noting that up to 45.1% of normal weight patients have high FTI/SMI ratios in the present study, suggesting that these high-risk patients might be overlooked in routine trauma care when using BMI as a risk assessment tool alone.

Our study had several limitations that should be considered. First, this was a single-center retrospective study with a relatively limited sample size, which limits the generalizability of our results to other populations, although we consecutively included abdominal trauma patients admitted to our hospital across the decade. Moreover, the relatively low numbers of underweight patients limited our ability to analyze the relationship between underweight and clinical outcomes in trauma patients, whereas it has been well demonstrated in prior studies [9,10]. Second, as with any observational study, the causalities of the relationship between the FTI/SMI ratio and outcomes were unable to be established, but its value as an independent predictor could be established and further guide clinical practice. Third, 167 patients were excluded due to unavailability or poor image quality of CT scans within 10 days before or after admission to hospital, which might affect the results. However, no significant association was found between the availability of CT scans and BMI or outcome. Fourth, patients admitted within 10 days of trauma might not have the same BMI, FTI or SMI measurements as baseline due to prehospital treatment. However, most patients (73%) were admitted to hospital within three days of trauma, so we believed the impact would be minor. Finally, as we collected body composition measurements at baseline, dynamic changes in body composition during the clinical course were not considered. Future research is needed to investigate the time-dependent dynamic effect of the FTI/SMI ratio on clinical outcomes in patients with abdominal trauma.

## 5. Conclusions

In conclusion, the obesity paradox was not observed in patients with abdominal trauma. Patients with overweight and obesity have higher mortality rates than normal-weight patients. A higher FTI/SMI ratio was significantly associated with increased 28-day mortality and ICU length of stay, which could be used as a predictor of adverse outcomes in trauma patients. Our findings were particularly useful for patients with high FTI/SMI but normal weight measured by BMI, and early targeted interventions might improve prognosis in this subgroup.

## Figures and Tables

**Table 1:** Clinical characteristics and outcomes of abdominal trauma patients stratified by BMI and 28-day mortality of admission.

Characteristics	Overall (n = 328)	Underweight (n = 13)	Normal (n = 233)	Overweight (n = 60)	Obese (n = 22)	*p* Value	Low FTI/SMI (n = 164)	High FTI/SMI (n = 164)	*p* Value
**Demographics**									
Age, y	43 (31, 53)	32 (25, 60)	43 (29, 53)	47 (38, 51)	40 (32, 46)	0.259	40 (27, 51)	46 (36, 54)	0.001 *
Male	266 (81.1)	11 (84.6)	188 (80.7)	47 (78.3)	20 (90.9)	0.675	31 (18.9)	31 (18.9)	1.000
Smoking history	102 (31.1)	6 (46.2)	66 (28.3)	23 (38.3)	7 (31.8)	0.298	50 (30.5)	52 (31.7)	0.811
Alcohol consumption	91 (27.7)	4 (30.8)	56 (24.0)	21 (35.0)	12 (54.5)	0.083	36 (22.0)	55 (33.5)	0.019 *
Diabetes	23 (7.0)	0 (0)	18 (7.7)	4 (6.7)	1 (4.5)	0.971	6 (3.7)	17 (10.4)	0.017 *
Hypertension	61 (18.6)	2 (15.4)	43 (18.5)	12 (20.0)	4 (18.2)	0.991	23 (14.0)	38 (23.2)	0.033 *
**Body composition**									
BMI, kg/m^2^	22.2 (20.8, 24.0)	17.6 (17.3, 18.0)	21.6 (20.6, 22.6)	25.1 (25.0, 26.0)	29.4 (28.7, 30.9)	<0.001 *	21.7 (20.3, 23.0)	22.9 (21.3, 25.0)	<0.001 *
FTI/SMI	1.5 (0.9, 2.0)	0.7 (0.4, 1.0)	1.4 (0.8, 2.0)	1.6 (1.0, 2.1)	2.1 (1.8, 2.7)	<0.001 *	0.9 (0.5, 1.2)	2.0 (1.7, 2.4)	<0.001 *
VATI/SMI	0.8 (0.4, 1.1)	0.4 (0.2, 0.6)	0.7 (0.4, 1.1)	0.9 (0.5, 1.2)	1.3 (1.0, 1.5)	<0.001 *	0.4 (0.3, 0.6)	1.1 (1.0, 1.4)	<0.001 *
SATI/SMI	0.6 (0.4, 0.9)	0.3 (0.2, 0.4)	0.6 (0.4, 0.9)	0.7 (0.5, 0.9)	0.9 (0.7, 1.3)	<0.001 *	0.4 (0.2, 0.6)	0.8 (0.7, 1.1)	<0.001 *
**Clinical parameters**									
GCS < 9	46 (14.0)	1 (7.7)	29 (12.4)	9 (15.0)	7 (31.8)	0.109	19 (11.6)	27 (16.5)	0.203
SBP < 90 mmHg	16 (4.9)	1 (7.7)	10 (4.3)	4 (6.7)	1 (4.5)	0.577	6 (3.7)	10 (6.1)	0.305
HR > 120 beats/min	32 (9.8)	0 (0)	27 (11.6)	4 (6.7)	1 (4.5)	0.473	14 (8.5)	18 (11.0)	0.457
RR > 20 beats/min	63 (19.2)	1 (7.7)	47 (20.2)	11 (18.3)	4 (18.2)	0.818	28 (17.1)	35 (21.3)	0.327
Blunt trauma	301 (91.8)	13 (100.0)	212 (91.0)	56 (93.3)	20 (90.9)	0.832	154 (93.9)	147 (89.6)	0.160
ISS ≥ 16	241 (73.5)	8 (61.5)	161 (69.1)	53 (88.3)	19 (86.4)	0.008 *	111 (67.7)	130 (79.3)	0.017 *
AIS score head ≥ 3	54 (16.5)	3 (23.1)	34 (14.6)	13 (21.7)	4 (18.2)	0.458	25 (15.2)	29 (17.7)	0.551
AIS score thorax ≥ 3	144 (43.9)	5 (38.5)	97 (41.6)	31 (51.7)	11 (50.0)	0.485	61 (37.2)	83 (50.6)	0.014 *
AIS score abdomen ≥ 3	243 (74.1)	9 (69.2)	164 (70.4)	49 (81.7)	21 (95.5)	0.031 *	116 (70.7)	127 (77.4)	0.166
AIS score extremities ≥ 3	56 (17.1)	0 (0)	36 (15.5)	15 (25.0)	5 (22.7)	0.090	21 (12.8)	35 (21.3)	0.040 *
Laparotomy	161 (49.1)	5 (38.5)	110 (47.2)	35 (58.3)	11 (50.0)	0.395	75 (45.7)	86 (52.4)	0.224
**Outcomes**									
28-day mortality	21 (6.4)	0 (0)	10 (4.3)	8 (13.3)	3 (13.6)	0.025 *	5 (3.0)	16 (9.8)	0.013 *
Mechanical ventilation	101 (30.8)	4 (30.8)	65 (27.9)	21 (35.0)	11 (50.0)	0.156	40 (24.4)	61 (37.2)	0.012 *
ICU length of stay, d	5 (2, 10)	7 (3, 13)	4 (1, 10)	6 (2, 10)	6 (4, 32)	0.030 *	4 (1, 8)	6 (2, 13)	0.009 *
Hospital length of stay, d	14 (9, 23)	13 (9, 17)	14 (9, 22)	14 (9, 23)	20 (13, 34)	0.185	13 (9, 21)	15 (10, 24)	0.135

BMI, body mass index; SMI, skeletal muscle index; FTI, fat tissue index; VATI, visceral adipose tissue index; SATI, subcutaneous adipose tissue index; GCS, Glasgow Coma Scale; SBP, systolic blood pressure; HR, heart rate; RR, respiratory rate; ISS, injury severity score; AIS, abbreviated injury scale; ICU, intensive care unit. Data were shown as n (%) for categorical variables, and median (P25, P75) or mean (standard deviation, SD) for continuous variables. * *p* value < 0.05.

**Table 2:** Associations between different body composition indices and 28-day mortality, mechanical ventilation, and ICU length of stay ≥ 5 d in patients with abdominal trauma.

Body Composition	Unadjusted		Model 1		Model 2	
	OR (95% CI)	*p* Value	OR (95% CI)	*p* Value	OR (95% CI)	*p* Value
**28-day mortality**						
BMI (Overweight vs. Normal weight)	3.43 (1.25–9.12)	0.013 *	3.43 (1.24–9.26)	0.015 *	4.47 (1.40–14.97)	0.012 *
BMI (Obese vs. Normal weight)	3.52 (0.74–12.71)	0.072	4.04 (0.83–15.20)	0.051	6.56 (1.07–36.57)	0.032 *
FTI/SMI ^a^ (H vs. L)	3.44 (1.31–10.72)	0.019 *	3.15 (1.19–9.87)	0.030 *	3.06 (1.08–10.16)	0.046 *
VATI/SMI ^b^ (H vs. L)	2.09 (0.85–5.66)	0.121	1.81 (0.72–4.95)	0.221	1.64 (0.60–4.84)	0.346
SATI/SMI ^c^ (H vs. L)	3.44 (1.31–10.72)	0.019 *	3.31 (1.26–10.38)	0.023 *	3.33 (1.16–11.13)	0.034 *
**Mechanical ventilation**						
BMI (Overweight vs. Normal weight)	1.39 (0.75–2.52)	0.283	1.36 (0.73–2.48)	0.328	1.14 (0.53–2.40)	0.740
BMI (Obese vs. Normal weight)	2.58 (1.06–6.32)	0.035 *	2.70 (1.09–6.68)	0.030 *	1.85 (0.54–6.20)	0.318
FTI/SMI ^a^ (H vs. L)	1.84 (1.14–2.97)	0.012 *	1.71 (1.06–2.80)	0.030 *	1.46 (0.79–2.73)	0.227
VATI/SMI ^b^ (H vs. L)	2.48 (1.53–4.07)	<0.001 *	2.27 (1.39–3.78)	0.001 *	1.99 (1.05–3.82)	0.037 *
SATI/SMI ^c^ (H vs. L)	1.45 (0.91–2.34)	0.075	1.40 (0.87–2.27)	0.165	1.27 (0.69–2.35)	0.441
**ICU length of stay ≥ 5 d**						
BMI (Overweight vs. Normal weight)	1.68 (0.95–3.02)	0.079	1.69 (0.95–3.05)	0.079	1.46 (0.77–2.79)	0.247
BMI (Obese vs. Normal weight)	2.40 (0.97–6.47)	0.066	2.31 (0.93–6.26)	0.081	2.13 (0.76–6.35)	0.156
FTI/SMI ^a^ (H vs. L)	1.80 (1.17–2.80)	0.008 *	1.80 (1.15–2.82)	0.010 *	1.75 (1.06–2.91)	0.031 *
VATI/SMI ^b^ (H vs. L)	1.63 (1.06–2.53)	0.028 *	1.63 (1.04–2.57)	0.035 *	1.39 (0.83–2.33)	0.215
SATI/SMI ^c^ (H vs. L)	1.34 (0.87–2.07)	0.185	1.33 (0.86–2.07)	0.201	1.28 (0.79–2.10)	0.321

Model 1: Adjusted for sex and age. Model 2: Adjusted for sex, age, injury severity score ≥ 16, hypertension, diabetes, smoking history, alcohol consumption, heart rate > 120 beats/min, respiratory rate > 20 beats/min, systolic blood pressure < 90 mmHg, Glasgow Coma Scale score < 9 and laparotomy. ^a b c^ Sex-specific medians were used as the cutoff values for FTI/SMI (1.39 in men, 2.02 in women), VATI/SMI (0.75 in men, 0.85 in women) and SATI/SMI (0.54 in men, 1.17 in women) ratios, respectively. L, low; H, high; BMI, body mass index; SMI, skeletal muscle index; FTI, fat tissue index; VATI, visceral adipose tissue index; SATI, subcutaneous adipose tissue index; ICU, intensive care unit. * *p* value < 0.05.

## Data Availability

The datasets used and/or analyzed during the current study are not publicly available due to patient privacy concerns, but are available from the corresponding author on reasonable request.

## Supplementary Materials

The following supporting information can be downloaded at: https://www.mdpi.com/article/10.3390/jcm12041503/s1, Figure S1: Scatter plots of different body composition indexes in patients with abdominal trauma. (a): BMI and FTI; (b): BMI and SMI; (c): FTI and SMI; (d): BMI and SATI; (e): BMI and VATI; (f) SATI and VATI; Table S1: Association between abdominal fat distribution and 28-day mortality, mechanical ventilation and ICU length of stay ≥ 5 d in patients with abdominal trauma.
