# Supplementary material for: High Fat-to-Muscle Ratio Was Associated with Increased Clinical Severity in Patients with Abdominal Trauma
Source: J Clin Med. 2023 Feb 14;12(4):1503. doi: 10.3390/jcm12041503 (PMC9960721; doi:10.3390/jcm12041503)
Supplement: Supplementary file 1 [file jcm-12-01503-s001.zip › jcm-2173116-supplementary.pdf]

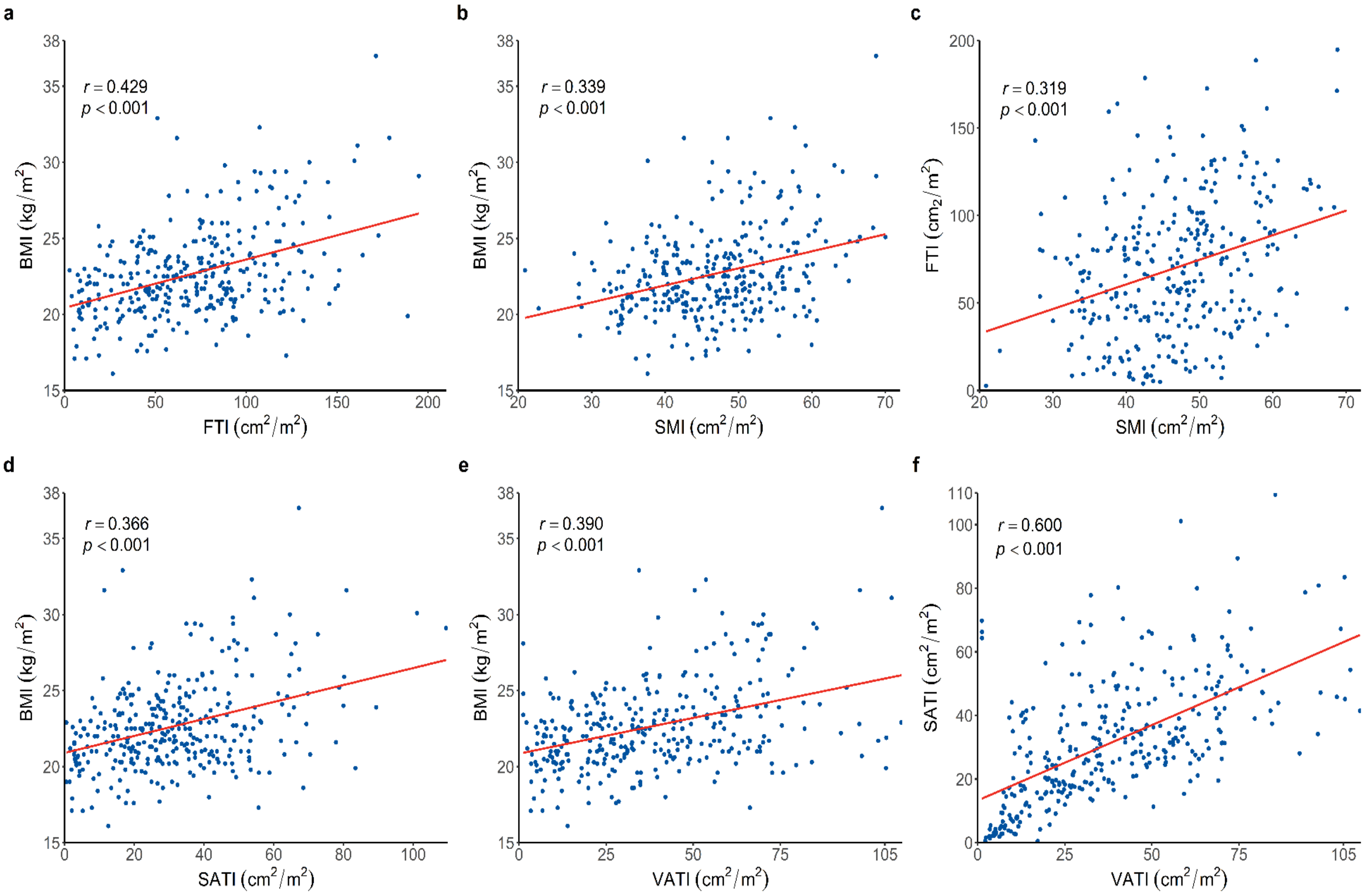

Figure S1 Scatter plots of different body composition indexes in patients with abdominal trauma. (a): BMI and FTI; (b): BMI and SMI; (c): FTI and SMI; (d): BMI and SATI; (e): BMI and VATI; (f) SATI and VATI. Pearson correlation coefficients and *P* values were provided. BMI, body mass index; FTI, fat tissue index; SMI, skeletal muscle index; SATI, subcutaneous adipose tissue index; VATI, visceral adipose tissue index.

Table S1 Association between abdominal fat distribution and 28-day mortality, mechanical ventilation and ICU length of stay ≥ 5 d in patients with abdominal trauma

Outcomes	Unadjusted		Model 1		Model 2	
	OR (95% CI)	*P* value	OR (95% CI)	*P* value	OR (95% CI)	*P* value
**Mortality**						
VATI/SATI[a] (H vs. L)	1.68 (0.69-4.35)	0.264	1.40 (0.56-3.73)	0.477	1.44 (0.55-3.99)	0.462
**Mechanical ventilation**						
VATI/SATI[a] (H vs. L)	1.37 (0.86-2.20)	0.189	1.20 (0.74-1.96)	0.464	0.99 (0.53-1.83)	0.962
**ICU length of stay ≥ 5 d**						
VATI/SATI[a] (H vs. L)	1.22 (0.79-1.88)	0.377	1.18 (0.75-1.86)	0.466	1.14 (0.69-1.90)	0.602

Model 1: Adjusted for sex and age. Model 2: Adjusted for sex, age, injury severity score ≥ 16, hypertension, diabetes, smoking history, alcohol consumption, heart rate>120 beats/min, respiratory rate>20 beats/min, systolic blood pressure < 90 mmHg, Glasgow Coma Scale score < 9 and laparotomy. [a] Sex-specific medians were used as the cutoff values for VATI/SATI (0.75 in men, 0.85 in women). VATI, visceral adipose tissue index; SATI, subcutaneous adipose tissue index; ICU, intensive care unit.
